# Intraoperative Heparin Resistance after Administration of Andexanet Alfa to Manage an Internal Iliac Artery Aneurysm Rupture: A Case Report

**DOI:** 10.3400/avd.avd.cr.23-00060

**Published:** 2023-10-11

**Authors:** Kanetsugu Nagao, Shigeyuki Yamashita, Rina Ebe, Norihito Naruto, Hisakatsu Ito, Saori Nagura, Toshio Doi, Kazuaki Fukahara, Naoki Yoshimura

**Affiliations:** 1 First Department of Surgery, University of Toyama, Toyama, Toyama, Japan; 2 Department of Radiology, University of Toyama, Toyama, Toyama, Japan; 3 Department of Anesthesia, University of Toyama, Toyama, Toyama, Japan

**Keywords:** andexanet alfa, heparin resistance, endovascular aneurysm repair

## Abstract

Antithrombotic agents are increasingly prescribed to older adults; however, they are associated with bleeding-related complications. We describe a case of intraoperative heparin resistance after administration of andexanet alfa (AA). An 81-year-old man was diagnosed with a ruptured internal iliac artery aneurysm. The patient required emergency endovascular aneurysm repair and was treated with AA because he was receiving apixaban. Despite high-dose intraoperative heparin administration, his activated coagulation time was not prolonged. Our findings suggest that AA should be administered with caution in patients experiencing potentially fatal bleeding (requiring surgical intervention) who are also receiving direct oral anticoagulants.

## Introduction

Antithrombotic agents are increasingly being prescribed to older adults with cardiovascular and cerebrovascular diseases. However, these agents are associated with bleeding-related complications, particularly intracranial and gastrointestinal bleeding, which can be fatal. Andexanet alfa (AA), an oral direct factor Xa inhibitor antagonist, is used to control severe bleeding in patients receiving direct oral anticoagulants.^1)^ However, heparin resistance during emergency surgery has been reported after AA administration.^2,3)^ We report a case of intraoperative heparin resistance due to preoperative AA administration in a patient with a ruptured internal iliac artery aneurysm.

### Case Report

**Figure 1:**
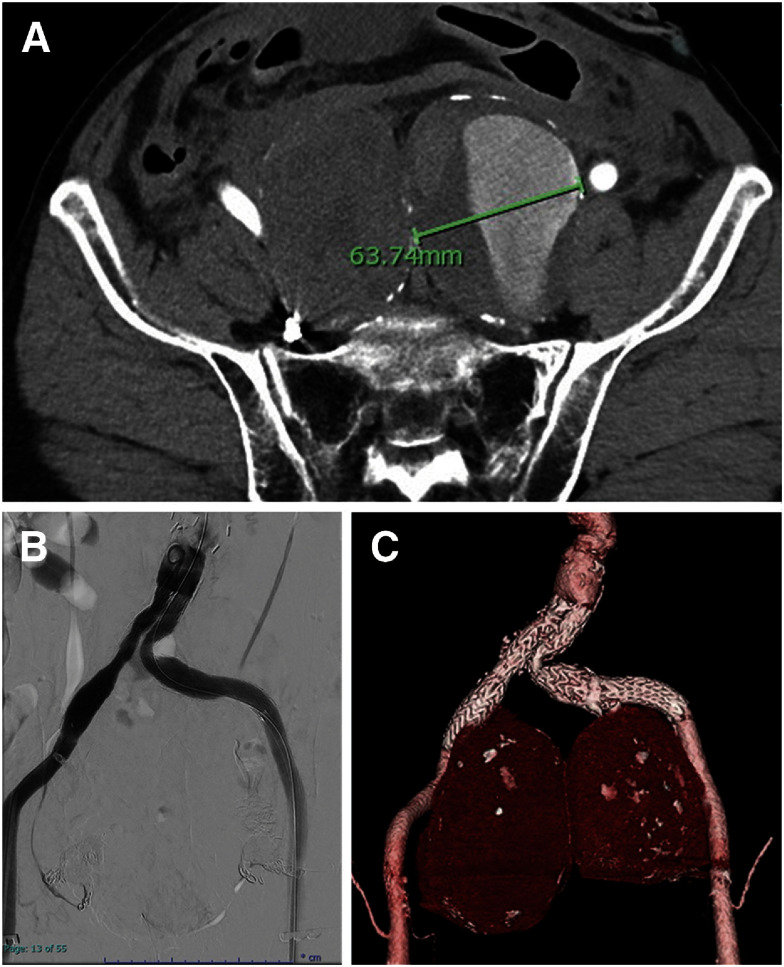
Fig. 1 (**A**) Preoperative computed tomography image. (**B**) Intraoperative final angiographic portrait. (**C**) Postoperative computed tomography image (3D reconstruction). 3D: three-dimensional

The patient was an 81-year-old man who was under outpatient observation for an unruptured left internal iliac artery aneurysm as well as postoperative abdominal aortic and right internal iliac artery aneurysms. He had previously received apixaban (10 mg/day) for his history of deep vein thrombosis. In August 2022, he experienced a prolonged bowel movement in the morning and abdominal fullness. The patient was diagnosed with constipation and monitored at home for approximately half a day; however, his condition did not improve and he was referred to our hospital for emergency care. Upon arrival at our hospital, his heart rate and blood pressure were 106 beats/min and 93/72 mmHg, respectively, and he was in a pre-shock state. His abdomen was distended and soft. Computed tomography revealed a ruptured left internal iliac artery aneurysm for which he had previously been under outpatient observation. The aneurysm had a maximum diameter of 63 mm and was accompanied by a mural thrombus and retroperitoneal hematoma. The upper and lower innominate arteries remained open, and the rupture site was located proximally to the aneurysm (**Fig. 1A**). The patient was referred for emergency surgery. AA was administered in the emergency room using the low-dose method, after which the patient was transferred to the operating room. He was 163.2 cm tall and weighed 66.5 kg. Preoperative blood test results (prior to AA administration) showed a hemoglobin level of 10.0 g/dL, platelet count of 186,000/μL, albumin level of 3.1 g/dL, C-reactive protein level of 4.40 mg/dL, antithrombin III activity level of 83%, and fibrin degradation product level of 11.1 μg/mL.

**Figure 2:**
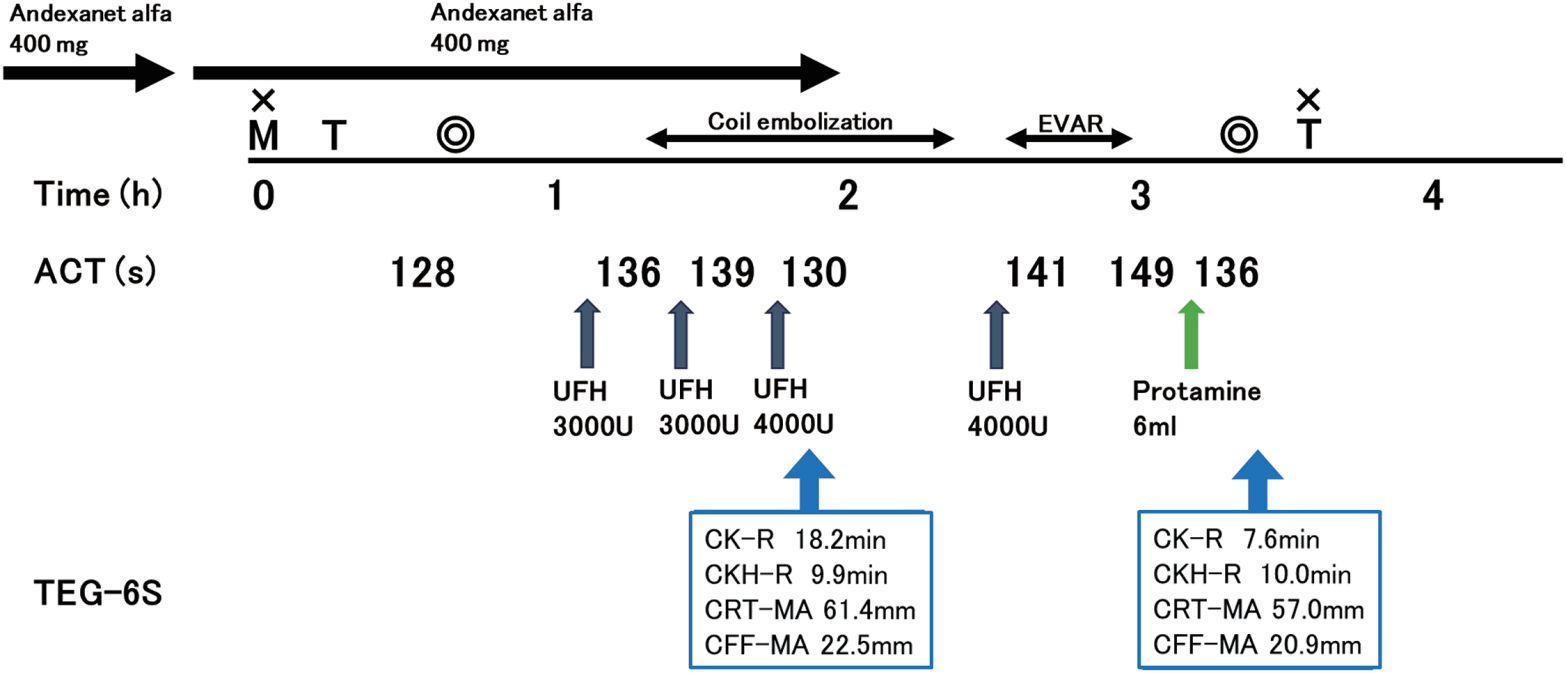
Fig. 2 Infusion of AA and heparin during surgery under general anesthesia. ACT is 149 s following infusion of 14,000 U UFH. TEG parameters following infusion of 10,000 U UFH and protamine are shown here. AA: andexanet alfa; ACT: activated coagulation time; citrated kaolin (CK); citrated kaolin with heparinase (CKH); citrated rapid TEG (CRT); citrated functional fibrinogen (CFF); UFH: unfractionated heparin; TEG: thrombelastographs; EVAR: endovascular aneurysm repair; R: reaction time; MA: maximum amplitude; ×: start/end of anesthesia; M: start of mask ventilation; T: intubation/extubation; ⦾: start/end of surgery

One day after symptom onset, selective coil embolization of the left superior and inferior popliteal arteries was performed under general anesthesia through a left common femoral artery approach, and endovascular aneurysm repair was performed through the left leg (**Figs. 1B** and **1C**). The operation time was 144 min, and the intraoperative blood loss was 50 g; two units of red blood cells were transfused. The baseline intraoperative activated coagulation time (ACT) was 128 s. The ACT only prolonged to 130 s after 10,000 U of unfractionated heparin (UFH) was administered. We suspected AA-induced heparin resistance; therefore, AA administration was discontinued, and 4000 U of additional UFH was administered. Despite this, the ACT did not become sufficiently prolonged (only achieved a maximum of 149 s). Thus, an intraoperative evaluation with thromboelastography (TEG; TEG 6s; Haemonetics Corp., Boston, MA, USA) was performed. Citrated kaolin with heparinase reaction time (CKH-R) involves the use of kaolin and heparinase reagent to evaluate endogenous coagulability, excluding the effects of heparin. Citrated functional fibrinogen (CFF) utilizes tissue factor and GPIIb/IIIa inhibitor to inhibit platelet coagulation and evaluate fibrinogen-only clot strength (**Fig. 2**). Although his ACT showed poor prolongation, the citrated kaolin reaction time (CK-R)–CKH-R difference was prolonged to 8.3 min, and the heparin was judged to be effective. No coagulopathy was observed intraoperatively, and the surgery was successfully completed.

Postoperatively, the patient awoke normally and was extubated and admitted to the intensive care unit. The patient was transferred to the general ward on the first postoperative day and to the rehabilitation ward on the 15th postoperative day. No thrombotic events occurred during the postoperative period.

## Discussion

Intraoperative heparin resistance is defined as an inability to achieve an ACT of 400–480 s with a UFH dose of ≥500 U/kg.^4)^ Heparin resistance is caused by abnormalities in the coagulation system and platelets, as well as by certain drugs. The effects of AA on heparin resistance have been recently reported.^5)^

AA, a recombinant modified human factor Xa decoy protein, reverses the effect of direct factor Xa inhibitors by binding to these small molecules with higher affinity than the endogenous factor Xa.^6)^ A previous case report used blood draw data and heparin doses to assess heparin sensitivity.^7)^ Upon examining this patient’s sensitivity to heparin in previous surgeries requiring heparinization, we noted that heparin sensitivity was clearly decreased in this case (**Table 1**). The patient’s history revealed no new potential causes of heparin resistance; therefore, heparin resistance was attributed to AA administration.

**Table 1:** Blood test results, heparin dose, and heparin sensitivity in previous surgeries requiring heparinization

Surgery (year)	Y-graft replacement (2007)		EVAR (2016)		EVAR (2022)	
	Preoperative	Postoperative	Preoperative	Postoperative	Preoperative	Postoperative
Platelet count (10^4^/μL)	21.8	17.4	21.1	20.5	10.8	10.4
Fibrinogen level (mg/dL)	387	347	399	416	251	304
Antithrombin III activity level (%)	102	76	94	95	83	68
Activated coagulation time (s)						
Baseline	132		130		128	
After heparin administration	187		300		149	
Heparin sensitivity index (s/units/kg)	1.15		2.15		0.10	
Heparin dose (U/kg)	48		79		211	

EVAR: endovascular aneurysm repair

TEG is reportedly superior to ACT for detecting UFH at blood concentrations <0.7 U/mL.^8)^ In this case, the ACT was not significantly prolonged. However, TEG was simultaneously performed, and the results showed that the effects of UFH were consistent with those noted in the operative field. Although there is no clear standard value of CK-R for UFH control, maintaining CK-R–CKH-R within 5–15 min has been reported to be ideal for extracorporeal membrane oxygenation (ECMO) management; thus, this range can be considered as a target. In similar cases where immediate TEG testing is not feasible, administration of an antithrombin 3 (AT III) preparation is recommended.^9)^ Further, although AA-induced heparin resistance only had minor effects in the present case, it could cause serious complications in surgical cases where systemic heparinization is necessary. This case was unusual because we performed a TEG evaluation in addition to ACT; moreover, despite the ACT not being prolonged, heparin was effective based on the TEG results. This case suggests that TEG may be useful in determining the efficacy of UFH after AA administration.

## Conclusion

Herein, we report a case of AA-induced heparin resistance. Our findings suggest that AA should be administered with caution in patients receiving direct oral anticoagulants who have fatal bleeding requiring surgical intervention. TEG can be useful for determining the efficacy of UFH after AA administration.

## Informed Consent

Informed consent was obtained from the patient regarding publication of the manuscript.

## Disclosure Statement

All authors have no conflicts of interest.

## Author Contributions

Study conception: KN

Data collection: All authors

Analysis: All authors

Investigation: KN, SY, and TD

Writing: KN

Critical review and revision: All authors

Final approval of the article: All authors

Accountability for all aspects of the work: All authors
